# Ciprofloxacin prophylaxis during haematopoietic cell transplantation: a role for use in patients with germ cell tumours?

**DOI:** 10.1099/jmm.0.001847

**Published:** 2024-06-26

**Authors:** Konstantinos Kavallieros, Ioannis Baltas, Giannis Konstantinou, Eirini Koutoumanou, Malick M. Gibani, Mark Gilchrist, Frances Davies, Jiri Pavlu

**Affiliations:** 1 Faculty of Medicine, Imperial College London, London, UK; 2 Department of Infection, Immunity and Inflammation, Institute of Child Health, University College London, London, UK; 3 Department of Haematology, Hammersmith Hospital, Imperial College Healthcare NHS Trust, London, UK; 4 Population, Policy & Practice Research and Teaching Department, Great Ormond Street Institute of Child Health, University College London, London, UK; 5 Department of Infectious Disease, Faculty of Medicine, Imperial College London, London, UK; 6 Department of Infectious Disease, Imperial College NHS Healthcare Trust, St Mary's Hospital, London, UK

**Keywords:** allografts, antimicrobial drug resistance, autografts, ciprofloxacin, hematopoietic stem cell transplantation, neoplasms, germ cell and embryonal

## Abstract

**Introduction.** Fluoroquinolone prophylaxis during haematopoietic cell transplantation (HCT) can lead to antimicrobial resistance (AMR). Identifying the groups of patients that have the highest likelihood of benefiting from prophylactic antimicrobials is important for antimicrobial stewardship (AMS).

**Hypothesis.** We aimed to identify groups of HCT recipients that have the highest likelihood of benefiting from prophylactic fluroquinolones.

**Methods.** All admissions for HCT in a tertiary centre between January 2020 and December 2022 (*N* = 400) were retrospectively studied. Allogeneic HCT (allo-HCT) recipients had prophylaxis with ciprofloxacin during the chemotherapy-induced neutropenia, while autologous HCT (auto-HCT) recipients did not. Bacteraemias were recorded when non-contaminant bacterial pathogens were isolated in blood cultures.

**Results.** Allo-HCT was performed for 43.3 % (173/400) of patients and auto-HCT was performed for 56.7 % (227/400). A bacteraemia was documented in 28.3 % (113/400) of cases. Allo-HCT recipients were more likely to have a Gram-positive bacteraemia (20.8%, 36/173, vs 10.1%, 23/227, *P* = 0.03), while a difference was not observed for Gram-negative bacteraemias (18.5%, 32/173 vs 18.1%, 41/227, *P* = 0.91). Among auto-HCT recipients not receiving ciprofloxacin prophylaxis, patients with germ cell tumours had the highest probability (*P* for trend 0.09) of recording any bacteraemia (43.5%, 10/23) followed by patients with lymphomas (32.5%, 13/40), other auto-HCT indications (22.2%, 2/9), multiple myeloma (22.1%, 29/131) and multiple sclerosis (12.5%, 3/24). The higher number of bacteraemias in patients with germ cell tumours was primarily driven by Gram-negative pathogens.

**Conclusions.** Ciprofloxacin prophylaxis was associated with a reduced incidence of Gram-negative bacteraemias in allo-HCT recipients. Auto-HCT recipients due to germ cell tumours, not receiving ciprofloxacin prophylaxis, recorded the highest incidence of bacteraemias and represent a possible target group for this intervention.

## Background

Bacteraemia is a significant complication of post-haematopoietic cell transplantation (HCT), contributing to both morbidity and mortality [1]. Fluoroquinolone prophylaxis during the chemotherapy-induced neutropenic period has been found to reduce infection occurrence in this group of patients [2]. However, fluoroquinolones are broad-spectrum antimicrobials, and their prolonged use promotes antimicrobial resistance (AMR) [3]. Additionally, recent Medicines and Healthcare Products Regulatory Agency (MHRA) warnings suggest that any potential benefits must be weighed against the rare risk of potentially long-lasting or irreversible side effects associated with these drugs [4]. Therefore, their use in HCT remains controversial, causing significant variation in practice worldwide. This can range from using fluoroquinolone prophylaxis for all HCT patient groups to not using it at all. In centres where prophylaxis is utilized, it is generally prioritized for allogeneic HCT (allo-HCT) recipients who carry an increased risk of infection due to the nature of the underlying malignancy, prolonged periods of neutropenia and graft-versus-host disease (GvHD) [5]. On the contrary, the use of fluoroquinolone prophylaxis in autologous HCT (auto-HCT) is less well established and might not be appropriate for all patient groups. Identifying high-risk groups within auto-HCT recipients who would benefit most from fluoroquinolone prophylaxis is important for antimicrobial stewardship (AMS).

In this article, we report the incidence of bacteraemia among different HCT populations in a large specialist centre in London, UK and assess the effectiveness of fluoroquinolone prophylaxis. Our aim was to identify patient groups within the auto-HCT population that would benefit most from fluoroquinolone prophylaxis.

## Methods

### Participants

Study participants included all patients admitted to Hammersmith Hospital, Imperial College Healthcare NHS Trust, London, UK, for HCT between 1 January 2020 and 31 December 2022. The list of patients was sourced from the local transplant registry; no patients admitted for HCT during this time period were excluded. Hammersmith Hospital is a 350-bed specialist tertiary referral hospital, specializing in haematology and transplant services, accredited by the Joint Accreditation Committee ISCT-Europe (JACIE) and European Society for Blood and Marrow Transplantation (EBMT). The hospital offers both matched unrelated donors, matched siblings and haploidentical HCT.

With regards to transplant procedures, all patients were admitted to the hospital in dedicated HCT wards, separated from the rest of the hospital’s clinical areas and nursed in High Efficiency Particulate Air-filtered positive pressure ventilation isolation side rooms. They remained inpatients from the start of conditioning chemotherapy until engraftment (absolute neutrophil count higher than 0.5×10^9^/L, sustained >20×10^9^/L platelets and haemoglobin >80 g/L, free of transfusion requirements), or resolution of adverse events, whichever was later. The hospital HCT protocols mandate primary antibacterial prophylaxis with ciprofloxacin for patients receiving allo-HCT while neutropenic, whereas auto-HCT recipients do not receive any anti-bacterial prophylaxis. Antifungal, antiviral and anti-pneumocystis/anti-toxoplasma prophylaxis is also offered as per EBMT guidelines [6]. In short, during the chemotherapy-induced neutropenic period, voriconazole or posaconazole is given to allo-HCT recipients as well as auto-HCT recipients with germ cell tumours, while the remaining auto-HCT recipients receive fluconazole. Aciclovir prophylaxis is given for five weeks from the day of HCT for allo-HCT recipients but only during the chemotherapy-induced neutropenic period for auto-HCT recipients. Co-trimoxazole anti-Pneumocystis and anti-Toxoplasma prophylaxis is given to all HCT, until the day of HCT and then resumed post-engraftment five weeks after HCT.

### Definitions, data sources and measurement

Data were collected from computerized medical records. Demographics collected included patient ethnicity, recorded as White, Asian, Black or other, and patient comorbidities, as per the International Severe Acute Respiratory and Emerging Infections Consortium [7]. The definition of neutropenia was an absolute neutrophil count of ≤0.5×10^9^ cells/L. A bacteraemia was recorded when a bacterial pathogen was isolated from a blood culture that did not fit the criteria for contamination. Contamination was determined by the growth of a common commensal organism in a single blood culture, which was not isolated again in repeat blood cultures [8]. A polymicrobial infection was recorded when there was growth of >1 pathogen in the same culture or different cultures taken <48 h apart.

### Statistical analysis

Data were analysed using Statistical Package for Social Sciences (SPSS) v.29 (IBM Corp.). Continuous variables were compared using the Student’s t, Mann–Witney U and Kruskal–Wallis tests, while Pearson’s Chi-squared test was used for categorical variables, as appropriate. Ten thousand bootstrap samples were used to calculate 95% confidence intervals (CIs), and the significance level was set at 0.05.

## Results

### Cohort characteristics

Between 1 January 2020 and 31 December 2022, there were 400 HCT admissions for 372 unique patients (HCT indications and conditioning regimes are shown in Tables S1 and S2, available in the online version of this article). Auto-HCT was performed in 56.7% (227/400) of admissions, while allo-HCT was performed in 43.3% (173/400). The two groups were comparable in baseline demographics and comorbidity status (Table 1) and have been described with regard to outcomes and antimicrobial utilization elsewhere [9]. In short, allo-HCT recipients had significantly longer hospital admissions [36 days (interquartile range [IQR]: 31–43.5) vs 22 days (IQR: 19–26), *P*<0.001] and neutropenic periods [19 days (IQR: 14–26) vs 8 days (IQR: 7–10), *P*<0.001]. Allo-HCT recipients had higher cumulative exposure to all antimicrobials and ciprofloxacin but lower exposure to piperacillin-tazobactam, meropenem, aminoglycosides and glycopeptides [9]. Overall mortality during the HCT admission was 4.5% (18/400) and deaths were primarily recorded in allo-HCT recipients (17/18 cases, 94.4%).

**Table 1.** Study participant characteristics

	All participants (*N* = 400)	Allo-HCT (*N* = 173)	Auto-HCT (*N* = 227)	*P*
**Demographics**				
Age	55 (43–62)	53 (42–60.5)	55 (44–63)	0.08
Sex				
Male	236 (59%)	112 (64.7%)	124 (54.6%)	0.05
Female	164 (41%)	61 (35.3%)	103 (45.4%)	
Ethnicity				
White	262 (65.5%)	114 (65.9%)	148 (65.2%)	0.48
Asian	93 (23.2%)	44 (25.4 %)	49 (21.6%)	
Black	22 (5.5%)	7 (4 %)	15 (6.6%)	
Other	23 (5.8%)	8 (4.6%)	15 (6.6%)	
Body mass index	26.6 (23.5–30.1)	26.3 (23.4–28.9)	27 (23.5–30.6)	0.12
**Comorbidities**				
Diabetes mellitus	44 (11%)	20 (11.6%)	24 (10.6%)	0.75
HIV	4 (1%)	0 (0%)	4 (1.8%)	0.08
Obesity	105 (26.3%)	35 (20.2%)	70 (30.8%)	0.02
Renal disease	26 (6.5%)	8 (4.6%)	18 (7.9%)	0.18
Renal dialysis	3 (0.8%)	1 (0.6%)	2 (0.9%)	0,73
Respiratory disease	7 (1.8%)	4 (2.3%)	3 (1.3%)	0.45
Asthma	36 (9%)	20 (11.6%)	16 (7%)	0.12
Cardiac disease	50 (12.5%)	16 (9.2%)	34 (15%)	0.09
Liver disease	10 (2.5%)	4 (2.3%)	6 (2.6%)	0.83
Neurological disease	29 (7.2%)	2 (1.2%)	27 (11.9%)	<0.001
Solid Neoplasm	7 (1.8%)	5 (2.9%)	2 (0.9%)	0.13
Rheumatological disease	8 (2%)	5 (2.9%)	3 (1.3%)	0.27
Karnofsky score	100 (100–100)	100 (90–100)	100 (90–100)	0.35
**Outcomes**				
Bacteraemia – all	113 (28.3%)	56 (32.4%)	57 (25.1%)	0.11
Bacteraemia – Gram-negative	73 (18.3%)	32 (18.5%)	41 (18.1%)	0.91
Bacteraemia – Gram-positive	59 (14.8%)	36 (20.8%)	23 (10.1%)	0.03
Neutropenia length (days)	11 (7–19)	19 (14–26)	8 (7–10)	<0.001
Length of stay in the hospital (days)	27 (21–36)	36 (31–43.5)	22 (19–26)	<0.001
Intensive care admission	32 (8%)	19 (11%)	13 (5.7%)	0.06
Death as inpatient	18 (4.5%)	17 (9.8%)	1 (0.4%)	<0.001

Continuous variables are presented as median (IQR), categorical variables as *N* (%). Allo-HCT: allogeneic haematopoietic cell transplantation; auto-HCT: autologous haematopoietic cell transplantation.

### Bacteraemias during HCT

**Fig. 1.**
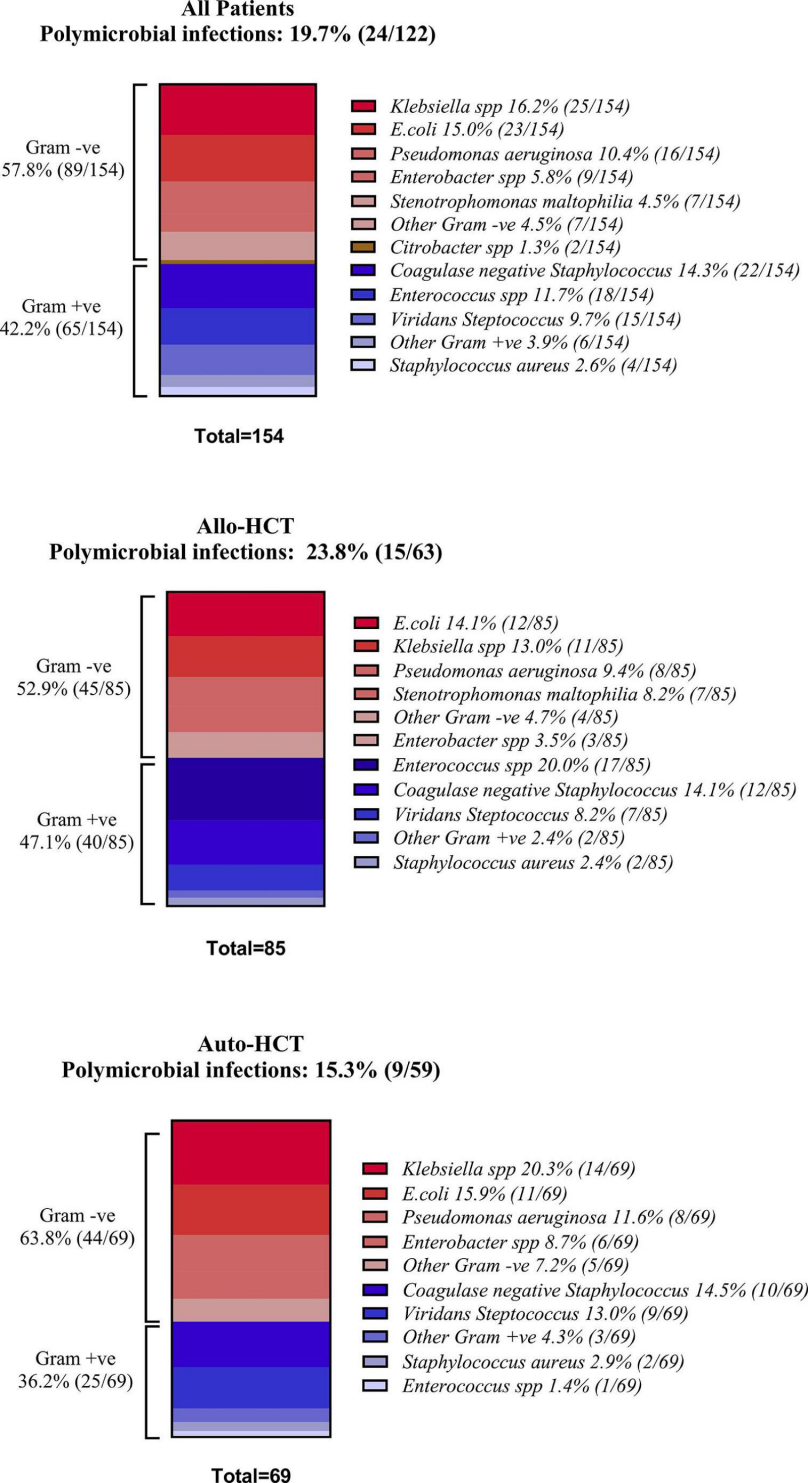
Causes of bacteraemia in the entire cohort and by transplant type. All strains reported were isolated from blood cultures and did not meet the criteria for contamination. Polymicrobial infections refer to the growth of more than one pathogen in the same culture or in different cultures taken within 48 h for the same infectious syndrome. Gram +ve: Gram-positive and Gram −ve: Gram-negative.

Bacteraemias were recorded in 28.3 % (113/400) of admissions during 122 unique infection episodes (some patients had more than one bacteraemia within the same admission). A total of 154 pathogens were isolated: 57.8 % (89/154) Gram-negative and 42.2 % (65/154) Gram-positive (Fig. 1). *Klebsiella* spp. (16.2% 25/154), *Escherichia coli* (15%, 23/154) and *Pseudomonas aeruginosa* (10.4%, 16/154) were the leading causes of Gram-negative bacteraemia, while *Coagulase-negative Staphylococcus* (14.3%, 22/154), *Enterococcus* spp. (11.7%, 18/154) and viridans streptococci (9.7%, 15/154) were the leading causes of Gram-positive bacteraemia. In total, 19.7% (24/122) of all bacteraemias were polymicrobial. Outcomes did not differ between monomicrobial and polymicrobial infections.

Overall, there was no statistically significant difference between the percentage of patients who developed bacteraemias between allo-HCT and auto-HCT recipients (32.4%, 56/173 vs 25.1%, 57/223, *P* = 0.11, respectively). However, allo-HCT recipients were significantly more likely to develop Gram-positive bacteraemia compared to auto-HCT recipients (20.8%, 36/173 vs 10.1%, 23/227, *P* = 0.003); no such difference was observed for Gram-negative bacteraemias (18.5%, 32/173 vs 18.1%, 41/227, respectively, *P* = 0.91), possibly secondary to the ciprofloxacin prophylaxis.

With regards to AMR, 35.5% (22/62) of all Enterobacterales isolated were extended-spectrum beta-lactamase (ESBL)-producers, while 33.9 % (21/62) were resistant to ciprofloxacin. There was significant overlap between ESBL production and ciprofloxacin resistance (90.9 % of cases, 20/22). Allo-HCT recipients were significantly more likely to isolate both ESBL-producing Enterobacterales (65.5%, 19/29 vs 9.1%, 3/33, *P*<0.001) as well as ciprofloxacin-resistant Enterobacterales (65.5%, 19/29 vs 6.1%, 2/33, *P*<0.001). On the contrary, all 16 *Pseudomonas aeruginosa* isolates identified (8 isolates in each group) were susceptible to ciprofloxacin and none exhibited a difficult-to-treat resistance phenotype.

### Outcomes by indication for auto-HCT

**Fig. 2.**
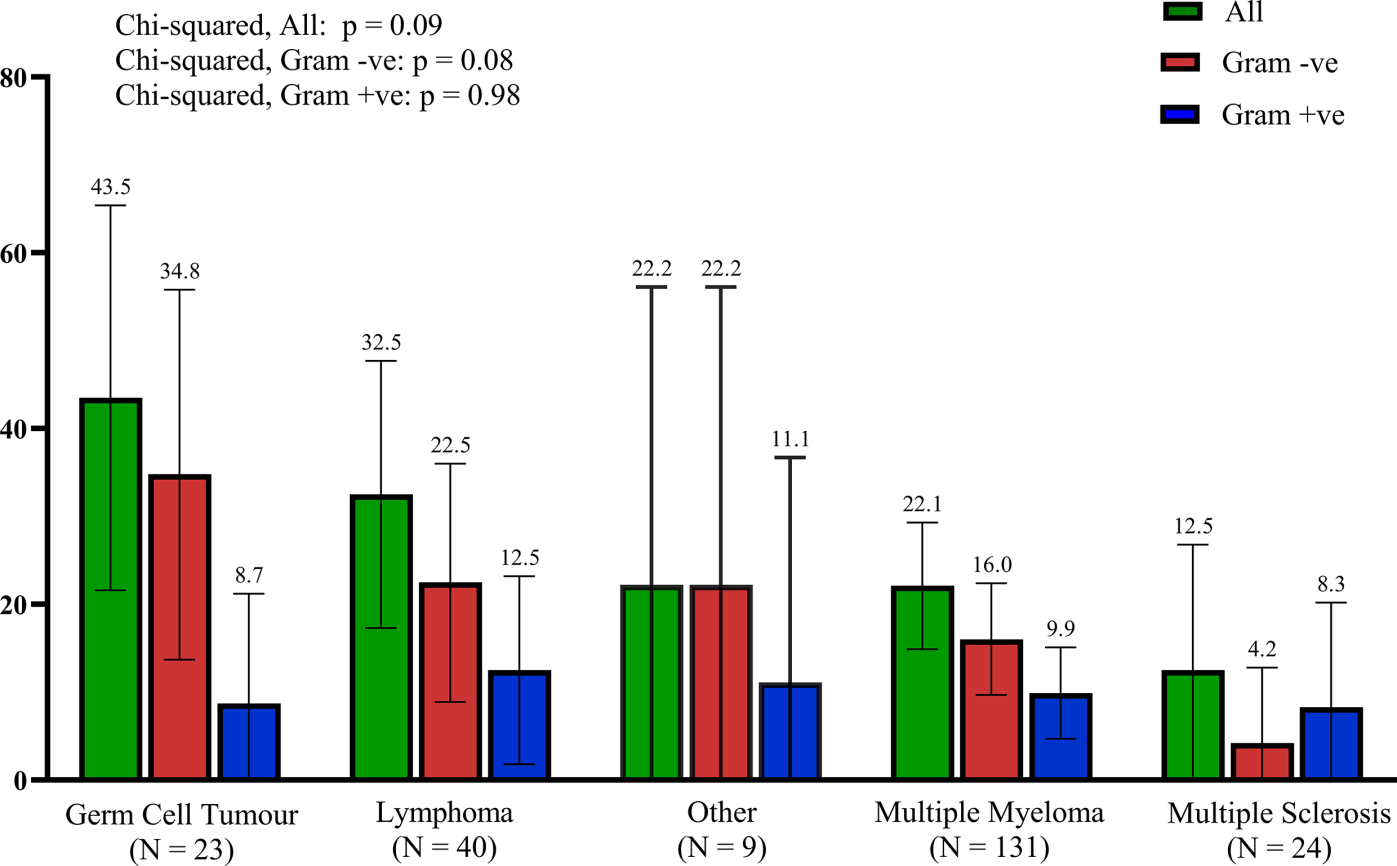
Percentage of admission episodes with documented bacteraemia (any, Gram-positive, Gram-negative) by auto-HCT indication type. None of the patients received fluoroquinolone prophylaxis during the chemotherapy-induced neutropenic period. Other (*N* = 9) include 3/9 beta-thalassemia; 3/9 POEMS syndrome; 1/9 acute promyelocytic leukaemia; 1/9 amyloidosis; 1/9 chronic myeloid leukaemia. Gram +ve: Gram-positive and Gram −ve: Gram-negative.

The most common indications for auto-HCT were multiple myeloma (57.8%, 131/227), lymphomas (17.6%, 40/227), multiple sclerosis (10.1%, 23/227) and germ cell tumours (10.1%, 23/227). None of the auto-HCT recipients received ciprofloxacin prophylaxis during the study period. Among auto-HCT recipients (Fig. 2), patients with germ cell tumours had the highest probability (*P* for trend 0.09) of recording a bacteraemia during their admission (43.5 %, 10/23, 95 % CI: 22.7–64.5), followed by patients with lymphomas (13/40, 32.5 % 95 % CI: 18.2–47.2), other auto-HCT indications (2/9, 22.2 % 95 % CI: 0–57.1 %), multiple myeloma (29/131, 22.1 % 95 % CI: 15.3–29.4) and multiple sclerosis (3/24, 12.5 % 95 % CI: 0.0–27.8). The higher number of bacteraemias in patients with germ cell tumours was primarily driven by Gram-negative pathogens (Fig. 2).

## Discussion

In this study, we have described a large cohort of HCT patients in a unique setting where hospital policy dictates that only patients undergoing allo-HCT receive fluoroquinolone prophylaxis during the chemotherapy-induced neutropenic period, while auto-HCT recipients do not. Our results indicate that this intervention led to a similar incidence of Gram-negative but not Gram-positive bacteraemia in allo-HCT recipients compared to auto-HCT recipients. This is surprising, as the former are at a higher risk of bacterial infections [10]. The rates of bacteraemia and pathogens identified were consistent with the current literature [3]. Mortality remained low in auto-HCT recipients. If ciprofloxacin prophylaxis is considered during auto-HCT, we found that patients with germ cell tumours have the highest rates of bacteraemia and should be prioritized for this intervention.

Our results are important, as they facilitate targeted use of antimicrobials in line with AMS. Not all auto-HCT indications are associated with the same infection risk, and germ cell tumour patients are an understudied group due to the rarity of the condition. Indeed, most studies investigating infections after auto-HCT describe patients with lymphomas and multiple myeloma [11,14]. Our large cohort from a specialist referral centre allowed us to showcase that auto-HCT recipients with germ cell tumours had a higher risk of bacteraemia compared to these traditional transplant indications. Therefore, in centres where ciprofloxacin prophylaxis is offered to auto-HCT recipients, our data suggest that germ cell tumour patients should be prioritized. On the contrary, ciprofloxacin prophylaxis might be a lower priority in other auto-HCT indications like multiple myeloma, balancing the risks and benefits of AMR versus morbidity and mortality from bacteraemia. This is particularly evident from our resistance results, which suggest a very high percentage (approximately 65%) of ESBL-producing and fluoroquinolone-resistant infections in allo-HCT recipients on ciprofloxacin prophylaxis. This was in stark contrast to infections in auto-HCT recipients (<10% resistance), as well as country-level data suggesting 15% of Enterobacterales in England are ESBL-producers or resistant to ciprofloxacin [15].

The main limitation of our study is the retrospective, single-centre design, which, although allowing for standardized treatment approaches between the parallel cohorts, makes conclusions challenging to extrapolate to other centres. It should also be noted that we are unable to differentiate between host characteristics and the effect of conditioning chemotherapy as the underlying factor for increased bacteraemia risk. Germ cell tumour patients in our cohort received carboplatin-based chemotherapy with etoposide and paclitaxel, which is known to be associated with significantly higher degrees of mucositis than melphalan- and cyclophosphamide-based regimes used in multiple myelomas and lymphomas, respectively [16]. This might explain the higher risk of bacteraemia in these patients and may indicate why ciprofloxacin prophylaxis in this group of patients might be beneficial. Yet, with a different conditioning regimen, the higher infection risk might no longer be observed.

In conclusion, the efficacy of fluoroquinolone prophylaxis in reducing Gram-negative infections must be balanced against the risk of AMR. To mitigate this risk, it is important to identify high-risk patient groups who will benefit most from prophylactic antimicrobials. High-quality prospective studies are needed to delineate which groups of HCT patients might benefit most from prophylaxis, enabling changes in clinical practice that reflect the increasing recognition of the challenges associated with AMR.

## supplementary material

10.1099/jmm.0.001847 Uncited Table S1.

## Abbreviations

allo-HCT: allogeneic HCT
AMR: antimicrobial resistance
AMS: antimicrobial stewardship
auto-HCT: autologous cell transplantation
CI: confidence interval
ESBL: extended-spectrum beta-lactamase
GvHD: graft-versus-host disease
HCT: haematopoietic cell transplantation
IQR: interquartile range
JACIE: joint accreditation committee ISCT-Europe and EBMT
MHRA: medicines and healthcare products regulatory agency
SPSS: statistical package for social sciences
