# Supplementary material for: Ciprofloxacin prophylaxis during haematopoietic cell transplantation: a role for use in patients with germ cell tumours?
Source: J Med Microbiol. 2024 Jun 26;73(6):001847. doi: 10.1099/jmm.0.001847 (PMC11316519; doi:10.1099/jmm.0.001847)
Supplement: Uncited Table S1. [file jmm-73-01847-s001.pdf]

Supplementary table 1: Indications for haematopoietic cell transplantation in study participants (N = 400)

Allogeneic haematopoietic cell transplantation (N = 173)	Autologous haematopoietic cell transplantation (N =227)
Acute myeloid leukaemia 37% (64/173)	Multiple myeloma 57.7% (131/227)
Non-Hodgkin's lymphoma 16.8% (29/173)	Non-Hodgkin's lymphoma 12.8% (29/227)
Myelodysplastic syndrome 10.4% (18/173)	Multiple sclerosis 10.6% (24/227)
Myelofibrosis 9.8% (17/173),	Germ cell tumour 10.1% (23/227)
Acute lymphoblastic leukaemia 9.2% (16/173),	Hodgkin's lymphoma 4.8% (11/227)
Chronic myeloid leukaemia 5.8% (10/173)	Other 4% (9/227)
Hodgkin's lymphoma 2.9% (5/173)	
Multiple myeloma 2.9% (5/173)	
Other 5.2% (9/173)	

Supplementary table 2: Hematopoietic cell transplantation conditioning regimens in study participants (N = 400)

	All (N =400)	Allo-HCT (N = 173)	Auto-HCT (N =227)
Cyclophosphamide	40.3% (161/400)	57.8% (100/173)	26.9% (61/227)
Mesna	38.3% (153/400)	53.2% (92/173)	26.9% (61/227)
Melphalan	37.5% (150/400)	8.7% (15/173)	59.5% (135/227)
Busulfan	31% (124/400)	67.1% (116/173)	3.5% (8/227)
Fludarabine	30% (120/400)	69.4% (120/173)	0% (0/227)
Methotrexate	23.5% (94/400)	54.3% (94/173)	0% (0/227)
Etoposide	18% (72/400)	6.9% (12/173	26.4% (60/227)
Alemtuzumab	16.3% (65/400)	37.6% (65/173)	0% (0/227)
Thiotepa	14.5% (58/400)	31.8% (55/173)	1.3% (3/227)
Lomustine	13% (52/400)	6.9% (12/173)	17.6% (40/227)
Cytarabine	13% (52/400)	8.7% (15/173)	16.3% (37/227)
Anti-thymocyte globulin	10.8% (43/400)	11% (19/173)	10.6% (24/227)
Carboplatin	5.8% (23/400)	0% (0/173)	10.1% (23/227)
Paclitaxel	5.3% (21/400)	0% (0/173)	9.3% (21/227)
Idarubicin	0.5% (2/400)	1.2% (2/173)	0% (0/227)
Rituximab	0.3% (1/400)	0.6% (1/173)	0% (0/173)
Total body irradiation	4.3% (17/400)	9.8% (17/173)	0% (0/227)
Total lymph node irradiation	3.3% (13/400)	7.5% (13/173)	0% (0/173)
Cranial irradiation	0.3% (1/400)	0.6% (1/173)	0% (0/173)
Reduced intensity chemotherapy	25% (100/400)	57.8% (100/173)	0% (0/227)
Haploidentical graft	11.8% (47/400)	27.2% (47/173)	0% (0/227)

Allo-HCT: Allogeneic haematopoietic cell transplantation; Auto-HCT: Autologous haematopoietic cell transplantation
